# Signals from the Brainstem Sleep/Wake Centers Regulate Behavioral Timing via the Circadian Clock

**DOI:** 10.1371/journal.pone.0070481

**Published:** 2013-08-12

**Authors:** Sabra M. Abbott, Jennifer M. Arnold, Qing Chang, Hai Miao, Nobutoshi Ota, Christine Cecala, Paul E. Gold, Jonathan V. Sweedler, Martha U. Gillette

**Affiliations:** 1 Department of Molecular & Integrative Physiology, University of Illinois at Urbana-Champaign, Urbana, Illinois, United States of America; 2 Cell & Developmental Biology, University of Illinois at Urbana-Champaign, Urbana, Illinois, United States of America; 3 Psychology, University of Illinois at Urbana-Champaign, Urbana, Illinois, United States of America; 4 Chemistry, University of Illinois at Urbana-Champaign, Urbana, Illinois, United States of America; 5 College of Medicine University of Illinois at Urbana-Champaign, Urbana, Illinois, United States of America; Kent State University, United States of America

## Abstract

Sleep-wake cycling is controlled by the complex interplay between two brain systems, one which controls vigilance state, regulating the transition between sleep and wake, and the other circadian, which communicates time-of-day. Together, they align sleep appropriately with energetic need and the day-night cycle. Neural circuits connect brain stem sites that regulate vigilance state with the suprachiasmatic nucleus (SCN), the master circadian clock, but the function of these connections has been unknown. Coupling discrete stimulation of pontine nuclei controlling vigilance state with analytical chemical measurements of intra-SCN microdialysates in mouse, we found significant neurotransmitter release at the SCN and, concomitantly, resetting of behavioral circadian rhythms. Depending upon stimulus conditions and time-of-day, SCN acetylcholine and/or glutamate levels were augmented and generated shifts of behavioral rhythms. These results establish modes of neurochemical communication from brain regions controlling vigilance state to the central circadian clock, with behavioral consequences. They suggest a basis for dynamic integration across brain systems that regulate vigilance states, and a potential vulnerability to altered communication in sleep disorders.

## Introduction

All individuals possess an internal circadian timing system, which regulates the appropriate timing of behavior within the 24 hour light-dark cycle. In mammals, the master circadian pacemaker is located within the suprachiasmastic nucleus (SCN) [1], [2]. A fundamental aspect of the circadian clock is its ability to respond to diverse signals from the body and the environment, in a time-of-day dependent manner, in order to adjust the timing of behaviors to adapt to these changes. One of the most well studied of these inputs is glutamate from the retinohypothalamic tract, which provides input regarding environmental light [3]. In addition, serotonergic inputs from the raphe are thought to provide input regarding activity state [4], norepinepherine inputs from the locus coeruleus are thought to provide inputs of arousal [5], [6], and melatonin provides inputs regarding dawn and dusk [7], [8], [9]. Less well understood, however, is the role of cholinergic inputs to the SCN.

Acetylcholine (ACh) was initially thought to provide the signal of light to the SCN. Early studies found that carbachol (a cholinergic agonist) injected into the lateral ventricles produced delays in the onset of activity, similar to those seen in response to light [10], and light pulses increased ACh levels at the SCN [11]. However depleting the brain stores of ACh with hemicholinium did not block the ability of the animal to respond to light [12], and applying ACh directly to the SCN either *in vitro* [13] or *in vivo* [14] did not produce a light-like pattern of circadian resetting. This raises the question of what role ACh plays in circadian resetting.

Some insight may be gained from looking at the location of potential cholinergic inputs. Neuroantomical tracing studies have demonstrated cholinergic projections to the SCN arising both in the basal forebrain from the nucleus basalis magnocellularis (NBM) and from the brainstem from the pedunculopontine tegmental nucleus (PPTg) and laterodorsal tegmental nucleus (LDTg) [15]. There have been several recent studies looking at the role of the basal forebrain cholinergic inputs to the SCN. Lesioning the cholinergic basal forebrain inputs attenuates the phase advances normally seen in response to light [16]. More recently it was reported that the cholinergic basal forebrain projections appear to be important for providing signals of cognitive entrainment to the SCN, specifically through tasks of sustained attention [17]. From this it is theorized that the forebrain cholinergic projections are important for the formation of time memories [18]. However, little is known about the role of the brainstem cholinergic inputs to the SCN.

The laterodorsal tegmental (LDTg) and pedunculopontine tegmental (PPTg) nuclei are two regions of a cell group of established importance for regulating vigilance state transitions [19]. The LDTg and PPTg send the majority of their efferent fibers to the thalamus [20], with additional efferents projecting from the LDTg to the ventral tegmental area [21], and from the PPTg to the substantia nigra, regulating dopamine release [22]. Lesions of the PPTg in cats decreases the amount of time spent in REM sleep [23], while stimulation of the LDTg increases the amount of time spent in REM [24]. Induction of REM sleep increases FOS levels in both the LDTg and PPTg [25]. It is possible, then, that the role of the brainstem cholinergic inputs to the SCN is to provide information regarding the vigilance state of the animal to the circadian clock.

Outputs from the SCN transmit time-of-day information that generates circadian oscillations in the functional state and physiology of brain stem regions that control sleep and waking [26], [27]. However, evidence of communication from the brain stem centers regulating sleep and wake, back to the circadian system has been less clear. Hamsters sleep-deprived during the latter part of their inactive period exhibit a significant advance in the onset of wheel-running activity [28], [29], thus homeostatic sleep-drive enhanced by sleep loss is able to influence the circadian system. Correlation between vigilance state and neuronal firing rate in the SCN suggests that brain regions regulating sleep/wake state transmit signals to the circadian system [30], [31]. Sources of this communication and functional consequences for circadian timekeeping are only beginning to be understood. Recent evidence has shown that the locus coeruleus plays an important role in providing information regarding arousal to the SCN [32], however, it is possible that the LDTg and PPTg are providing additional inputs regarding vigilance state to the circadian clock.

We examined the hypothesis that signals from brain regions that control vigilance state engage the circadian system. Despite anatomical evidence for neural projections connecting LDTg and PPTg neurons of the brain stem with the SCN [15], the neurochemical identity and functional context of this potential feedback loop have not been evaluated. Both direct and indirect cholinergic projections from these pontine nuclei innervate the SCN in nocturnal [15], [33] and diurnal [34] rodents. To test for functional communication, we stimulated discrete pontine sites in the mouse brain and measured two responses: changes at the SCN in levels of neurotransmitters from LDTg and PPTg neurons [15] that are stimulus-dependent, and changes in the phasing of behavioral circadian rhythms. Depending upon stimulus conditions, ACh and/or glutamate levels were found to be augmented in the SCN. Equivalent stimulations of the median raphe nucleus (MRN) had no effect on ACh or glutamate levels in SCN, but did increase SCN serotonin (5-HT). When effects of PPTg stimulation on behavioral circadian rhythms were assessed across the circadian cycle of animals in constant environments, parameters that caused glutamate release at the SCN produced significant shifts in behavioral rhythms, similar to those seen with direct glutamatergic stimulation of SCN brain slices [35] or *in vivo* [36]. Injection of glutamate into the PPTg in early subjective night also induced circadian delays, suggesting effects are due to local PPTg activation [37]. Our data provide the first evidence for a functional connection between brainstem cholinergic regions involved in regulating vigilance state and the circadian clock. These findings suggest that alterations in the vigilance state may, through these pathways, be able to feed back upon the circadian system to reset the SCN time-base, thereby maintaining the appropriate alignment between the circadian and sleep/wake systems.

## Results

**Figure 1:**
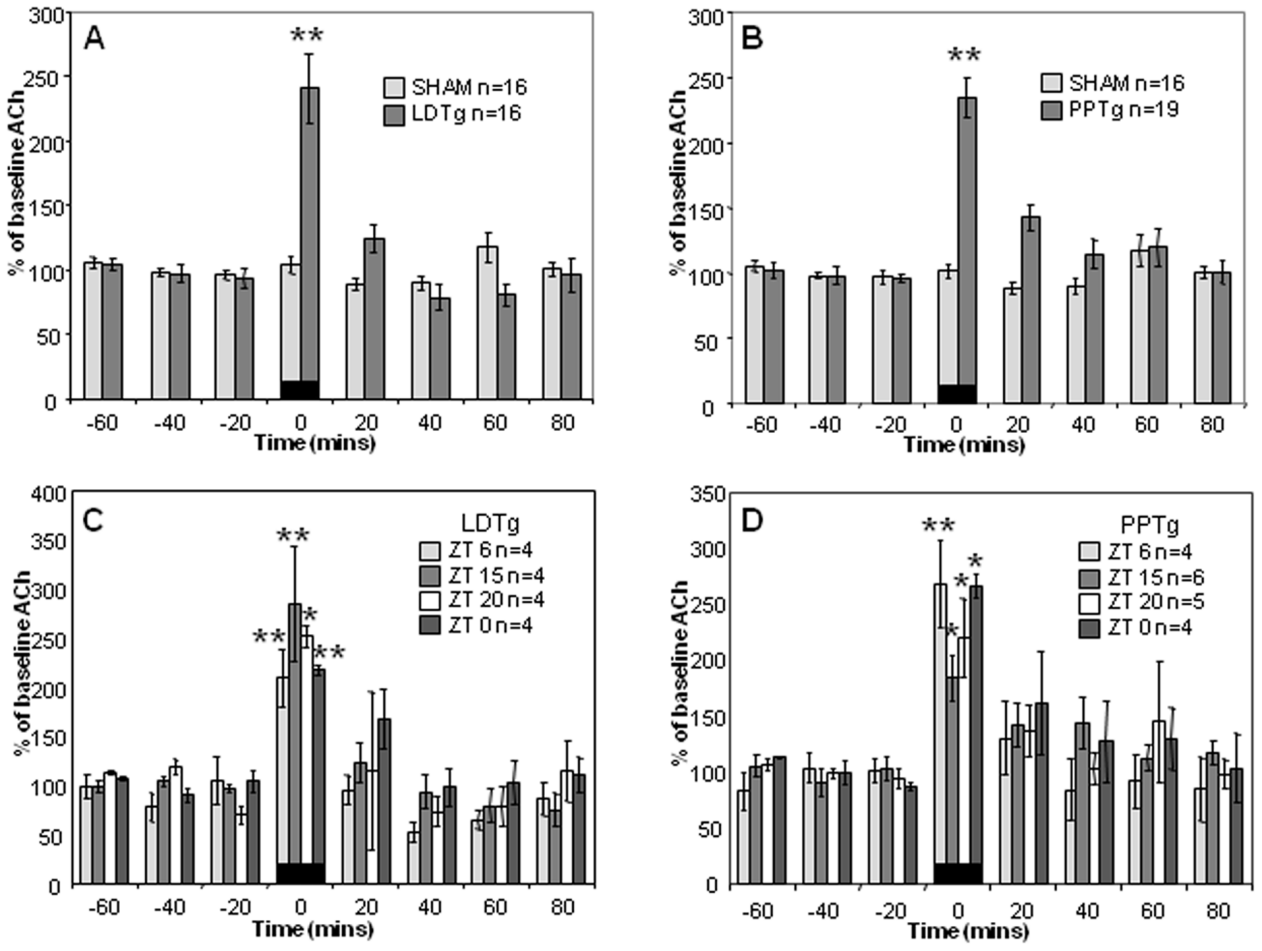
Electrical stimulation of LDTg or PPTg increases ACh at the SCN. Stimulating LDTg (**A**) or PPTg (**B**) at 150 µA, 10 Hz, 2-msec pulse duration causes significant increase in ACh levels in SCN dialysate. When LDTg (**C**) or PPTg (**D**) is stimulated at different times of day, SCN levels of ACh increased significantly at all times tested. The magnitude of response across different times of day is not significant. Black bars denote times of stimulation; significance level is marked (* = p<0.05, ** = p<0.001, Two-Way ANOVA with Holm-Sidak *post hoc* test). ACh, acetylcholine; LDTg, laterodorsal tegmental nucleus; PPTg, pedunculopontine tegmental nucleus; SCN, suprachiasmatic nucleus; ZT, Zeitgeber Time.

To investigate neurochemical communication between sleep/wake and circadian regulatory centers, we stimulated the LDTg or PPTg of freely-behaving mice on a 12hr:12hr light-dark schedule, while collecting microdialysate from the SCN for ACh analysis. Both LDTg and PPTg stimulation (150 μA, 10 Hz, 2-msec pulse duration) results in a significant increase in ACh release (240±25% and 231±18%, respectively, p<0.001, LDTg: treatment F(1, 30) = 15.3, p<0.001; time F (7, 105) = 9.98, p = 0.002; treatment × time interaction F(7, 105) = 15.8, p<0.001, PPTg: treatment F(1, 36) = 30.56, p<0.001, time F(7, 133) = 10.74, p<0.001, treatment × time interaction F(7, 133) = 10.92, p<0.001), when compared to baseline measurements (Figs. 1A, B). Sham conditions, in which animals are connected to the apparatus but not stimulated, show no difference from baseline (p = 1.000).

To determine whether the magnitude of ACh release depends on time-of-day, the data presented above were analyzed based on the time of stimulus application. Animals received the stimulus at 6, 15, 20 or 24/0 h after lights-on, Zeitgeber Time (ZT) 6, 15, 20 or 24/0. LDTg stimulation at each time-point significantly increases ACh release at the SCN, but differences among these time-points are not significant (ZT 6 = 208±29%, ZT 15 = 285±58% and ZT 24/0 = 217±30%, p<0.001, ZT 20 = 251±80%, p<0.05, treatment F(3, 12) = 17.96, p<0.001, ZT F(7, 24) = 1.60, p = 0.196, treatment × ZT F(21, 24) = 0.64, p = 0.88 Fig. 1C). Similar results are found following PPTg stimulation, with animals showing a significant increase in ACh release at the SCN during stimulation at all times of the day tested, but with no difference in magnitude of response seen as a factor of time-of-day of stimulation (ZT 15 = 184±23%, ZT 20 = 220±35% and ZT 24/0 = 266±46%, p<0.05, ZT 6 = 268±39%, p<0.001, treatment F(3, 16) = 14.71, p<0.001, ZT F(7, 32) = 0.659, p = 0.579, treatment × ZT F(21, 32) = 0.772, p = 0.748, Fig. 1D). This indicates that the ability of these pontine nuclei to respond to an electrical stimulus and cause ACh release at the SCN is not gated over the circadian cycle.

**Figure 2:**
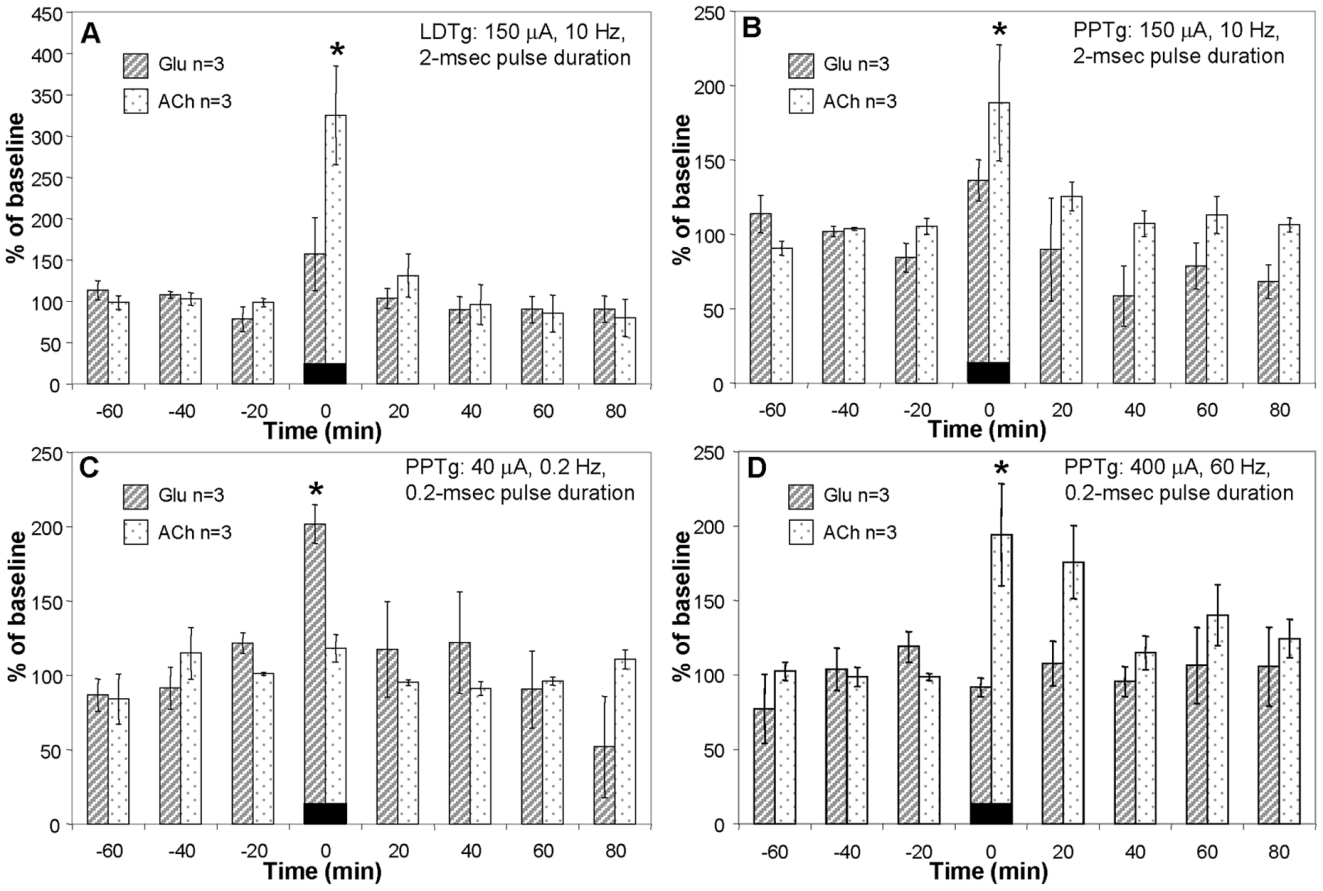
LDTg or PPTg stimulation increases glutamate or ACh at the SCN in early night. Following LDTg (**A**) or PPTg (**B**) stimulation with Condition 1 (150 µA, 10 Hz, 2-msec pulse duration), ACh significantly increases.PPTg stimulation with Condition 2 (**C**, 40 µA, 0.2 Hz, 0.2 msec pulse duration) significantly increases glutamate (Glu) release at the SCN. PPTg stimulation with Condition 3 (**D**, 400 µA, 60 Hz, 0.2 msec pulse duration, applied as a 1-sec train every min) significantly increases ACh release at the SCN. * indicates p<0.05 by Two-Way ANOVA with Holm-Sidak *post hoc* test.

To evaluate whether stimulation of the LDTg *vs.* PPTg results in differential neurotransmitter release, using the same settings we stimulated a second set of animals at ZT 15, this time co-analyzing releasate from each sample for both ACh and glutamate. Results indicate that following LDTg stimulation both ACh and glutamate levels increase significantly at the SCN (325±60% and 157±44%, respectively, time F(7, 16) = 6.544, p<0.001, releasate F(1, 4) = 1.057, p = 0.306, time × releasate interaction F(7, 1) = 1.16, p = 0.329, Fig. 2A).

Three stimulus conditions were used for the PPTg; parameters are detailed in the methods section. PPTg stimulation with Condition 1 significantly increases ACh (188±39%) but not glutamate (136±14%) release at the SCN (time F(7, 16) = 4.45, p<0.001, releasate F(1, 4) = 10.75, p = 0.001, time × releasate interaction F(7, 16) = 1.36, p = 0.228, Fig. 2B). Condition 2 causes a significant increase in glutamate (201±13%) but not ACh (118±9%) at the SCN (time F(7, 16) = 3.58, p = 0.006, releasate F(1, 4) = 0.98, p = 0.329, time × releasate interaction F(7, 16) = 2.64, p = 0.028, Fig. 2C). Condition 3, on the other hand, significantly increases ACh (194±34%) without affecting glutamate (91±6%) time F(7, 16) = 2.23, p = 0.06, releasate F(1, 4) = 11.42, p = 0.002, time × releasate interaction F(7, 16) = 2.38, p = 0.04, Fig. 2D). These findings suggest that the population of PPTg neurons that cause neurotransmitter release at the SCN may vary depending upon the context in which neurons are activated.

To evaluate whether neurotransmitter release at the SCN is specific to the brain region stimulated, we evaluated the response to stimulating the serotonergic median raphe nucleus (MRN), which does not synthesize ACh or glutamate. We analyzed SCN dialysate for serotonin (5-HT), as well as for ACh and glutamate. Stimulation of the median raphe significantly increases 5-HT at the SCN (Condition 3, 364±89%, p<0.001, 2-way ANOVA, time F(7, 40) = 1.74, p = 0.11, releasate F(2, 15) = 22.48, p<0.001, time × releasate interaction F(7, 40) = 2.83, p = 0.001), as anticipated, however does not affect ACh or glutamate levels (p = 1.000) (Fig. S1).

**Figure 3:**
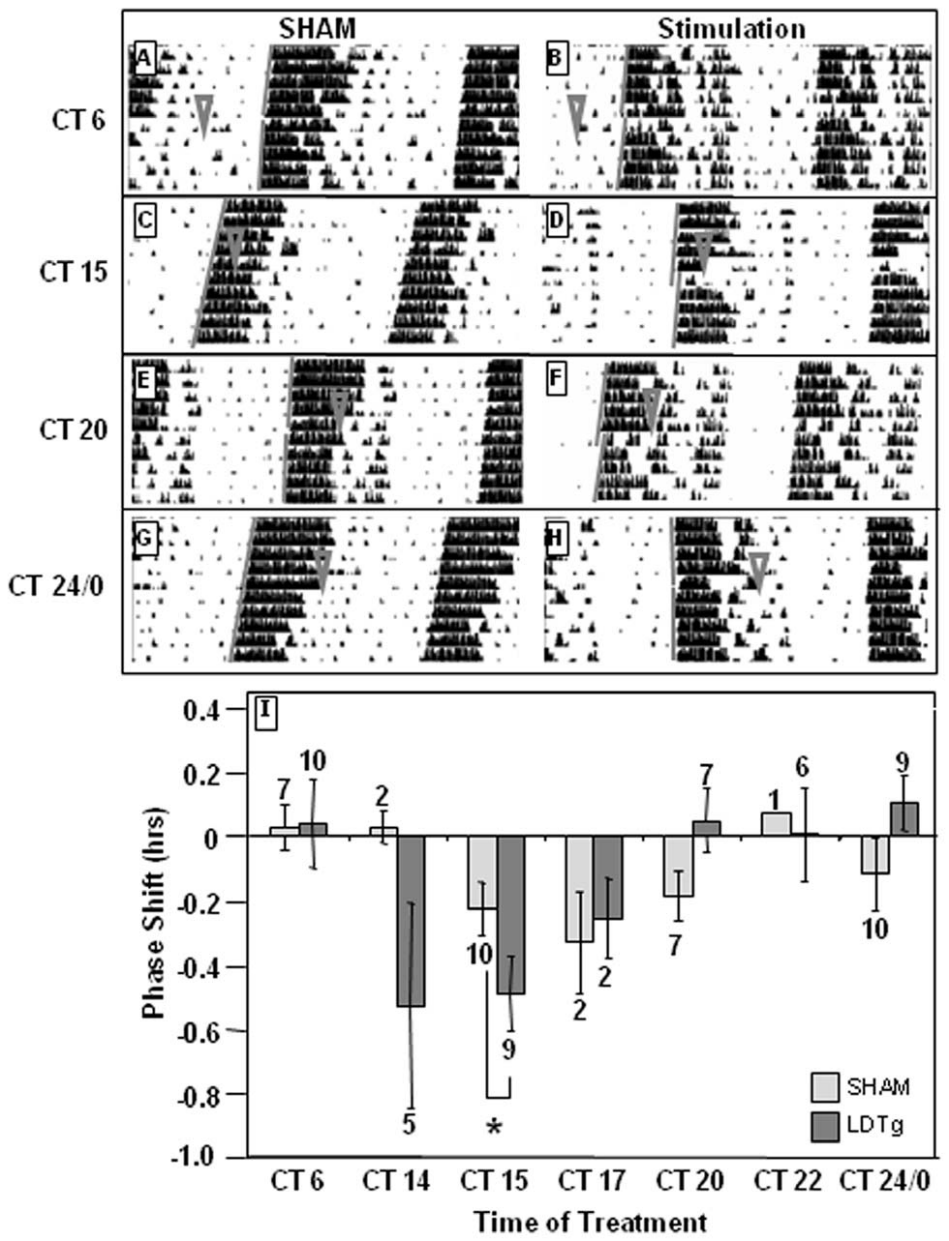
LDTg stimulation resets phasing of behavioral rhythms during subjective night. LDTg stimulation (150 µA, 10 Hz, 2-msec pulse duration, 20 min) at CT 14 and 15 (**D**) delays circadian behavioral rhythms while stimulation at CT 0 (**H**) advances circadian rhythms. **B**, **D**, **F**, and **H** are representative actograms following LDTg stimulations, while **A, C, E**, and **G** are representative actograms following sham stimulations. ∇ indicates treatment time. Gray lines indicate onset of activity before and after treatment. **I** depicts the average of all treatments. Numbers above or below each bar indicate number of animals in each treatment. * indicates p<0.05 by Two-Way ANOVA with Holm-Sidak *post hoc* test.

To determine whether these signals from the brainstem engage the circadian timekeeping mechanism in the SCN, we evaluated effects on phasing of wheel-running activity. Mice received LDTg stimulation (150 μA, 10 Hz, 2-msec pulse duration) in constant dark environments at circadian time (CT) 6, 14, 15, 17, 20, 22, or 24/0. Stimulation in early subjective night (15) causes a significant delay in locomotor rhythms (−0.54±0.11 hr, p = 0.02, CT F(6, 56) = 3.03, p = 0.01, treatment F (1, 18) = 0.273, p = 0.603, CT × treatment interaction F(6, 56) = 1.854, p = 0.1, Fig. 3).

**Figure 4:**
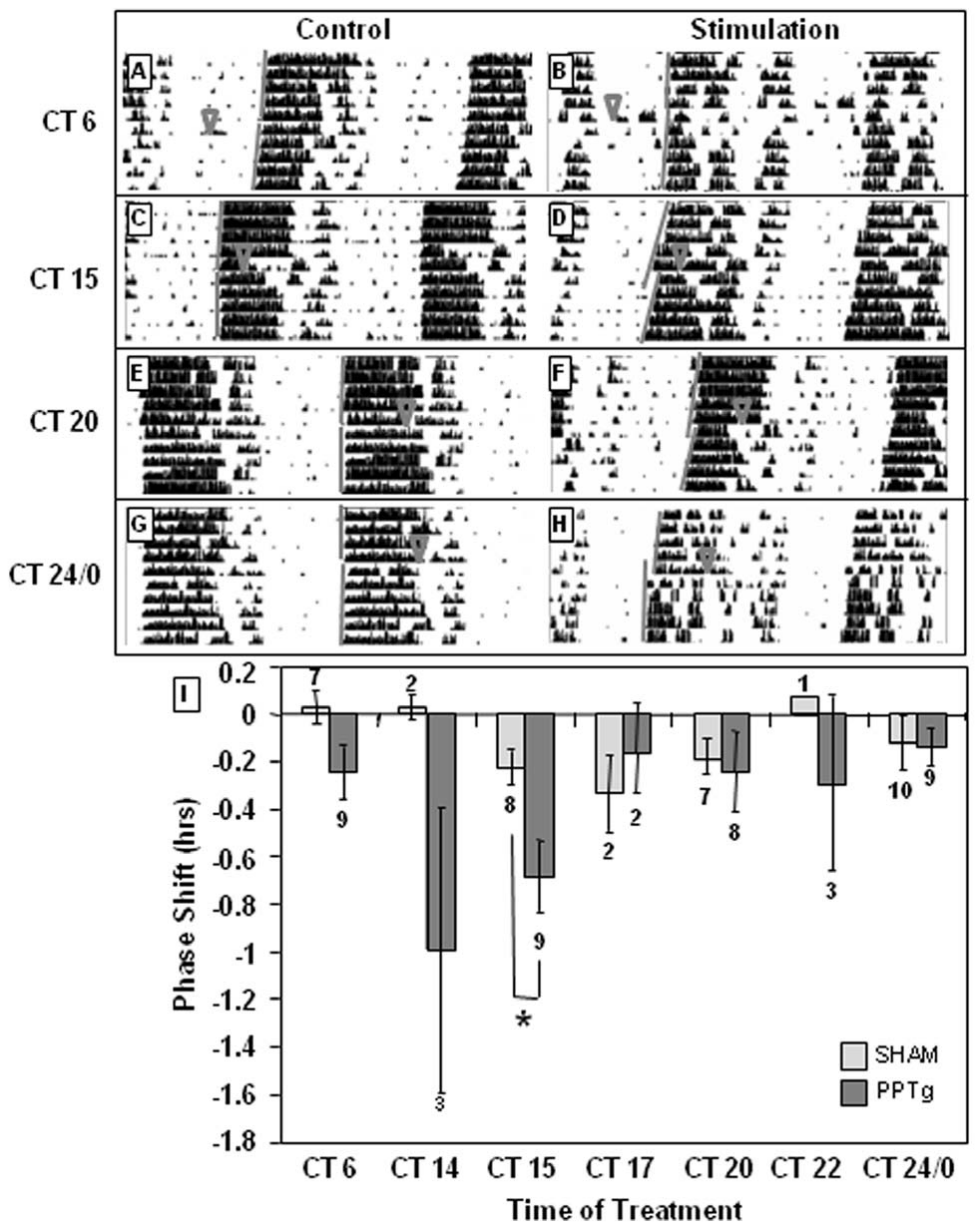
PPTg stimulation delays behavioral circadian rhythms only in early subjective night. PPTg stimulation (150 µA, 10 Hz, 2-msec pulse duration, 20 min) at CT 15 (**D**) delays circadian behavioral rhythms, whereas stimulation at other CTs does not significantly alter timing of circadian rhythms. **B, D, F**, and **H** are representative actograms following PPTg stimulations, while **A, C, E**, and **G** are representative actograms following sham stimulations. ∇ indicates treatment time. Gray lines indicate onset of activity before and after treatment. **I** depicts the average of all treatments. Number of animals in each treatment is indicated above or below the bar. * indicates p<0.05 by Two-Way ANOVA with Holm-Sidak *post hoc* test.

As with LDTg, stimulating the PPTg at CT 15 produces significant delays in circadian activity rhythms (−0.68 h, p = 0.01) when compared to sham stimulation (CT F(6, 56) = 1.99, p = 0.081, treatment F(1, 18) = 7.18, p = 0.009, CT × treatment interaction F(6, 56) = 1.75, p = 0.124 Fig. 4).

**Figure 5:**
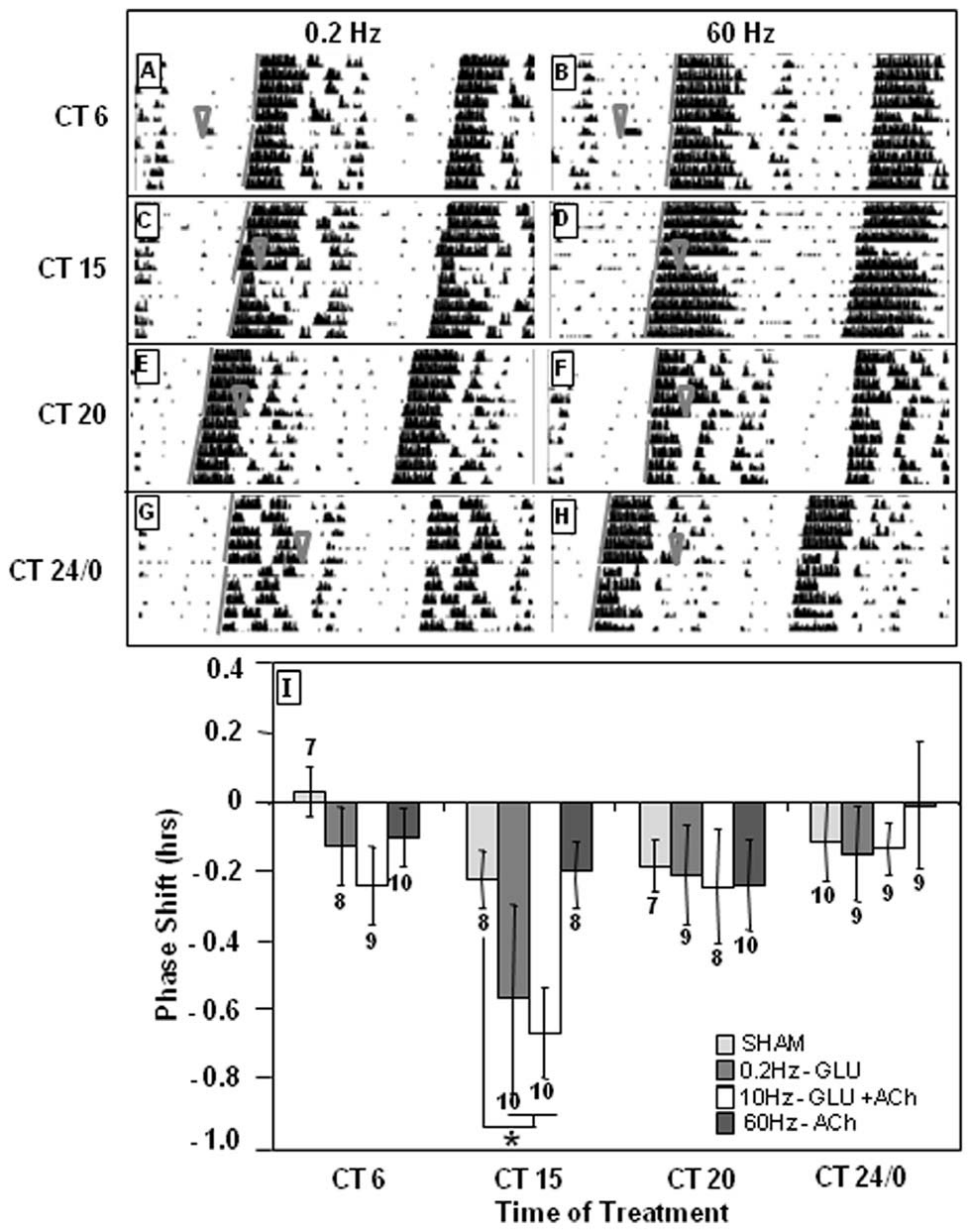
Early evening PPTg stimulus conditions favoring glutamate *vs.* ACh release at SCN alters behavioral rhythms. At CT 15, stimuli that increase glutamate (Glu) release (0.2 Hz, 40 µA, 0.2-msec pulse duration) or Glu and ACh release (10 Hz, 150 µA, 2-msec pulse duration) delay circadian behavioral rhythms. However, stimulus conditions that raise only ACh levels (60 Hz, 400 µA, 0.2-msec pulse duration) do not alter phasing of circadian rhythms at any time tested. **A, C, E**, and **G** are representative actograms from animals receiving 0.2-Hz stimulations. **B, D, F**, and **H** are representative actograms from animals receiving 60-Hz stimulations. ∇ indicates treatment time. Gray lines indicate onset of activity before and after treatments. **I** depicts the average of all treatments. Number of animals in each treatment appears above or below the bar. * indicates p<0.05 by Two-Way ANOVA with Holm-Sidak *post-hoc* test.

To determine whether the SCN undergoes circadian changes in sensitivity to these neurotransmitters, we applied the differential stimuli at points around the circadian cycle to animals under constant conditions. Animals received PPTg stimulation using the three conditions described above at CT 6, 15, 20 or 24/0. In early subjective night, at CT 15, significant delays are seen following Conditions 1 and 2 (p = 0.01) when compared to CT 6 or CT 0 (CT F(3, 36) = 4.66, p = 0.004, treatment F(3, 36) = 1.80, p = 0.15, Fig. 5). Phasing of behavioral rhythms is not altered following Condition 3 (p = 0.60). Together, these results show that activation of mesopontine tegmental nuclei significantly changes the phasing of wheel-running behaviors, an effect that is gated over the circadian cycle by clock state.

**Figure 6:**
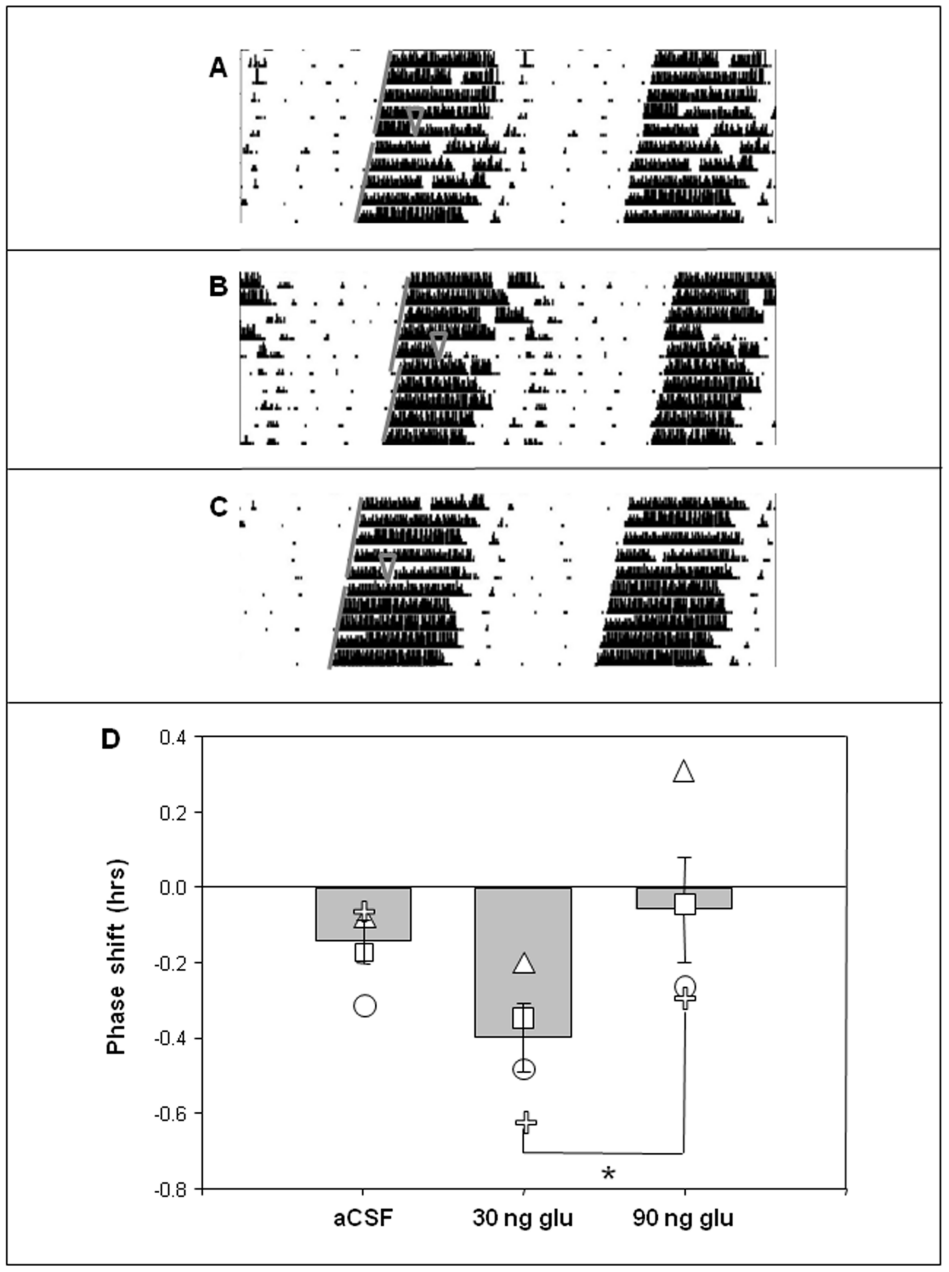
Chemical stimulation of the PPTg with glutamate delays behavioral circadian rhythms in a dose-dependent manner. Representative actograms of mice injected in PPTg at CT 15 with 30 ng/100 nl of glutamate (Glu) (**B**), but not aCSF (**A**) or 90 ng/100 nl Glu (**C**), showing a significant delay in behavioral rhythms. **D** presents the average response to each treatment. ∇ indicates treatment time. Gray lines indicate onset of activity before and after treatment. Small shapes indicate responses of individual animals. * p<0.05, paired *t*-test.

To assess whether chemical activation of the PPTg produces behavioral responses similar to electrical stimulation, and as a control for potential non-specific effects of electrical stimulation, glutamate was injected into the PPTg during early subjective night. Following injection with 30 ng/100 nl glutamate, significant phase delays in wheel-running behavior are observed (−0.4 hr, p<0.05, paired Student's *t*-test), whereas injection with either 90 ng/100 nl glutamate or aCSF has no effect on circadian rhythms (Fig. 6).

## Discussion

Our data provide evidence for functional communication between brain stem tegmental nuclei with established roles in regulating vigilance state transitions and the circadian clock in the SCN. Stimulation of the LDTg or PPTg of the C57BL/6J mouse causes stimulus-specific increases in ACh and/or glutamate at the SCN. In addition to altering neurotransmitter levels, activation of the LDTg or PPTg can induce functional changes in circadian behavior. The delays in phasing of behavioral rhythms in response to stimulation during the early subjective night are similar to those caused by direct glutamatergic stimulation of the SCN *in vitro* and *in vivo* [35], [36].

LDTg and PPTg both contain mixed populations of neurons [38]. Cholinergic and glutamatergic fibers project directly from brainstem to SCN [15] and also innervate intermediate cholinergic sites in basal forebrain [20]. Both ACh and glutamate appear in SCN dialysate, depending upon the stimulus applied to the brain stem sites. Glutamate levels increase in response to either LDTg or PPTg stimulation, although not to the same extent as ACh. While some stimulus conditions alter SCN levels of both neurotransmitters, it was possible to affect glutamate without increasing ACh and *vice versa*. This suggests that a mixed population of neurons projects from the PPTg to the SCN. While there is evidence for direct projections from brain stem to the SCN, cholinergic neurons of the basal forebrain also project to SCN. These forebrain projections have been reported to participate in circadian regulation of cognitive functions, such as selective attention, where they induce phase-advances in daytime [39]. While it is possible that our observed effects are the consequence of a relay that projects indirectly to the SCN rather than via direct communication, behavioral outcomes of the aggregate studies are more consistent with direct projections from the brain stem.

Circadian behavioral responses to PPTg stimulation are varied and reflect the neurotransmitter profile released at SCN. Intra-SCN injection of a glutamatergic agonist in the early night produces delays [36], while ACh produces advances [14]. Until now, however, the circadian response to the presence of both neurotransmitters simultaneously had not been examined. Two outcomes are possible: 1) the circadian response to the release of both neurotransmitters could be additive, so that the response to a delaying signal from glutamate and an advancing signal from ACh results in little or no net resetting, or 2) the circadian response could be hierarchical, with the response depending on the temporal sequence of activation or which signal is dominant, due to the relative efficacy of downstream signaling elements. In the latter cases, the net result may not differ from that stimulated by one or the other of the neurotransmitters alone. Our results indicate that stimulation parameters that cause an increase in ACh without glutamate produce little or no net resetting, while glutamate produces a response of comparable direction and magnitude to that observed if both ACh and glutamate are present. This indicates that the glutamate signal is dominant to ACh and, when this signal is activated, the presence of ACh does not alter the net outcome. It is noteworthy that the presence or absence of ACh does not have a significant effect on the net magnitude of the glutamatergic response. In retinal innervation of the SCN, co-release of pituitary adenylyl cyclase-activating peptide (PACAP) with glutamate is neuromodulatory [40].

It is noteworthy that, despite behavioral responses that vary based on circadian time of stimulation, we demonstrate that the amount of neurotransmitter released does not change with the time-of-day of stimulation. This suggests that the circadian responsiveness of the SCN is regulated post-synaptically, rather than pre-synaptically. This is consistent with our previous findings both *in vivo* [14] and *in vitro* [13] that application of equivalent doses of cholinergic agonists to the SCN at different circadian times produces very different circadian responses. Together these studies strongly support the temporal changes in SCN responsiveness to neurotransmitter stimulation as post-synaptically gated.

Given the potential limitations of electrical stimulation, including both tissue damage and potential activation of surrounding structures, our last set of studies activated the PPTg chemically. While there still the potential for surrounding tissue damage from the injection cannula, it is not extensive (Fig. S2). Using this technique, we demonstrate that chemical activation of the PPTg produces changes in circadian behavior similar to those seen with electrical stimulation. Injection of low concentrations of glutamate into the PPTg has been shown to induce REM-sleep in rats, while high concentrations of glutamate induce wakefulness [37]. Neurons within the rat PPTg are either REM-active or wake/REM-active [41], suggesting that the observed behavioral states may reflect activation of a specific population of neurons within the PPTg. Injection of a low concentration of glutamate into the PPTg of mice induces circadian resetting patterns similar to those observed following electrical stimulation of the PPTg, but higher concentrations of glutamate and control injections do not. While these results provide an interesting starting point, they are limited by the fact that it is not known how PPTg injections of glutamate affect the sleep-state of mice. In rats and cats, injection of low concentrations of glutamate, which are capable of resetting circadian behavioral rhythms, induce REM sleep, but not wake. As mice are nocturnal, and are normally in the middle of their active phase at this point, a signal indicating sleep would be predicted to alter circadian rhythms at this time, while a signal of wake would not. Of note, in looking at the actigraphy data, following injections of low, but not high, concentrations of glutamate, mice show a decrease in activity, suggesting they may be sleeping during this time period, however further studies in which the sleep state of the animal is recorded following the intra-PPTg glutamate injection will be needed to determine this.

Taken together, these studies demonstrate that signals from brainstem pontine tegmental regions to the SCN, either directly or indirectly, engage the circadian system. Activation of these pathways produces changes in neurotransmitter levels at the SCN and alters the phasing of circadian behavioral rhythms. A stimulus that resets the circadian clock may be an error signal reporting a temporal mismatch with other oscillatory systems. Pontine projections to the SCN originate from regions active during the sleep-wake cycle. Thus, neurotransmissions from the brain stem to the SCN at specific points in the day-night cycle may be interpreted as an error regarding arousal state in the context of time-of-day. Large bouts of sleep at abnormal times, as would be observed following sleep deprivation, might contribute to this error signal. The SCN generates output signals that inhibit locomotor activity [26], [27], so resetting the circadian clock after a period of sleep deprivation could decrease locomotor behavior, creating a state permissive for sleep and facilitating efficient recovery of sleep lost. Possible support of this hypothesis comes from a recent paper showing that 5 days of sleep restriction resulted in a delay in melatonin onset [42].

While there is substantial evidence that sleep need and time-of-day are important determinants of vigilance state, support for the notion that these processes interact functionally has been limited [43], [44]. Animals with a functional circadian clock recover more quickly from periods of sleep deprivation, suggesting that an interaction between the homeostatic and the circadian systems provides the most efficient means of maintaining homeostasis [45]. The firing rate of cells within the SCN is altered by sleep state [31] and sleep deprivation can affect clock gene expression in a time-of-day-dependent manner [46], evidence for bi-directional interaction between these two systems. Recently it has been demonstrated that the locus coeruleus provides an important signal regarding the arousal state of the animal to the SCN [32]. However, the present study is the first demonstrating a functional connection between brainstem pontine tegmental nuclei, known to be active during wake and REM sleep, and the SCN. Now that a functional connection between these two regions has been demonstrated, further experiments will need to be done to evaluate the sleep state of the animal that corresponds with these signals. Nonetheless, our current findings that activation of sleep/wake centers in the brainstem can cause resetting of circadian behavioral rhythms in a time-of-day- and state-dependent manner suggest previously unrecognized integration between these global brain systems, and identify new sites of potential vulnerability in disordered sleep.

## Materials and Methods

### Ethics Statement

All experimental procedures in this study were conducted under protocols approved by the Institutional Animal Care and Use Committee (IACUC) at the University of Illinois at Urbana-Champaign (Animal Welfare Assurance number A3118-01). All animal care and surgical procedures were conducted in full compliance with the recommendations outlined in the National Institutes of Health Guide for the Care and Use of Laboratory Animals. Survival surgeries were performed under sodium pentobarbital anesthesia with pre- and post-operative analgesia to minimize pain. Every effort was made in experimental planning to reduce the number of animals required for this study.

### Animals

Male C57BL/6J mice were obtained at 5 weeks of age from Jackson Laboratories (Bar Harbor, ME). Animals were housed and cared for as described in animal protocols in full compliance with NIH guidelines for the humane care and treatment of animals, approved by IACUC and supervised by the Division of Animal Resources at the University of Illinois at Urbana-Champaign.

### Surgical Procedures

Surgeries on mice (18–23 g) were performed ≥1 week after animals arrived. Mice were anesthetized with sodium pentobarbital (80 mg/kg i.p.). A microdialysis guide (CMA/7; CMA/Microdialysis; North Chelmsford, MA) was implanted in the SCN, and a bipolar stimulating electrode (MS303/1; Plastics One, Inc.; Roanoke, VA) was implanted into the LDTg, PPTg, or MRN using a stereotaxic apparatus (Stoelting; Wood Dale, IL). Coordinates based on *bregma* were: SCN, -0.1 mm AP, -0.1 mm ML, -4.0 mm DV; LDTg, -5.2 mm AP, -0.5 mm ML, -3.3 mm DV; PPTg, -4.3 mm AP, -1.8 mm ML, -3.7 mm DV; MRN, -3.8 mm AP, 0.0 ML, -4.8 DV. DV measurements were made from the dural surface, and coordinates were determined experimentally with the aid of a mouse brain atlas [47]. Cannulae were secured with small machine screws (0–80×1/16; Plastics One, Inc.; Roanoke, VA) and cranioplastic cement (Plastics One, Inc.; Roanoke, VA).

### Housing

Mice were housed individually in cages (30×17×22 cm) equipped with running wheels (14.5 cm dia.) that were placed inside ventilated light-tight wooden boxes (171×41×34 cm). Wheels were locked on the first day to minimize novel wheel-induced phase shifts, which can increase the time necessary for entrainment. For light-dark cycles, lights were kept at 100 lux for 12 h, followed by 12 h of darkness, regulated by computer. Up to 6 mice could be housed in each box. For experimental procedures, animals were placed in light-tight circadian activity monitoring systems (CAMS) and maintained in darkness under constant conditions [48].

### Electrical Stimulation

Three stimulus conditions were used for the PPTg: 1) 150 μA, 10 Hz, 2-msec pulse duration applied continuously for 20 min, as in previous experiments; 2) 40 μA, 0.2 Hz, 0.2-msec pulse duration continuously for 20 min; and 3) 400 μA, 60 Hz, 0.2-msec pulse duration applied as a 1-sec train every minute for 20 min. Conditions 2 and 3 have been shown previously to favor glutamate or ACh release, respectively, from these neurons [49], [50].

### ACh, Glutamate, and 5-HT Measurements

#### Microdialysis

For microdialysis procedures, animals were gently restrained, a microdialysis probe (CMA/7; CMA/Microdialysis; North Chelmsford, MA) was inserted into the dialysis guide aimed at the SCN and was attached to the animal using a head-block tether system (Harvard Apparatus; Holliston, MA). Connecting cables for stimulation (305-305 TT2; Plastics One, Inc.; Roanoke, VA) also were attached at this time. Animals were placed into a CAMS designated for stimulation experiments, where they were attached to fluid (Instech; Harvard Apparatus; Holliston, MA) and electronic swivels (Plastics One, Inc.; Roanoke, VA). Placing animals in CAMS for the duration of the experiment allowed experiments to be performed on a freely-behaving, undisturbed animal, under controlled environmental conditions.

Before each experiment, the microdialysis probe was placed in a standard solution containing ACh (20 nM) and choline (20 nM) for 20 min to determine the recovery rate of the probe. When inserted into the SCN, the probe was continuously perfused at a rate of 1 μl/min with artificial cerebral spinal fluid (aCSF; 0.13 M NaCl, 4 mM KCl, 0.75 mM NaH_2_PO_4_ (anhydrous), 2 mM Na_2_HPO_4_, 1 mM dextrose, 2 mM MgCl_2_, 1.7 mM CaCl_2_) containing 500 nm neostigmine, a cholinesterase inhibitor. Animals were allowed to equilibrate for at least 1 h before samples were collected. For experiments performed during the animal's dark period, the microdialyis probe was attached during the light period and animals were perfused with aCSF until 2 h before the first collection, at which point the perfusion fluid was switched to aCSF containing 500 nM neostigmine. This allowed all probes to be attached during the light period, while standardizing the length of time each animal was exposed to neostigmine. After ∼1 h for equilibration, baseline samples were collected every 20 min for 1 h. Animals were then stimulated, and microdialysis sample collection continued every 20 min for an additional 100 min. Samples were split for analysis of either ACh or glutamate content, and kept at −80°C until analysis. Samples collected for analysis of ACh, glutamate, and 5-HT content were split 3 ways. Following collection of the last sample, mice were sacrificed and brains harvested for histological verification of electrode and guide-cannula placement.

#### High Performance Liquid Chromatography (HPLC)

Microdialysis samples were analyzed for ACh content using HPLC in combination with an electrochemical detector (BAS; Bioanalytical Systems; West Lafayette, IN). Sample (5 μl) was injected using a low-dispersion injection valve with a 5 μl polyetheretherketone loop (Rheodyne model 9125-087). Samples were run through a mobile phase containing 50 mM Na_2_HPO_4_ (pH 8.5) and 0.5% Kathon (BAS P/N CF-2150) at a rate of 140 μl/min. Samples first passed through an ion-exchange microbore analytical column (BAS P/N MF-8904, 530×1 mm) followed by a microbore ACh/choline immobilized enzyme reactor (IMER) containing acetylcholinesterase (AChE) and choline oxidase (BAS P/N MF-8903, 50×1 mm). This series of columns separated ACh from choline, and converted it to H_2_O_2_. The concentration of H_2_O_2_ was measured using a horseradish peroxidase-coated 3 mm glass-carbon working electrode held at a 100 mV potential *vs.* a Ag/AgCl working electrode. ACh concentrations were determined by comparing peak heights of samples to peak heights of standard solutions and were then corrected based on the recovery values determined for the probe used for each experiment [51].

### Capillary Electrophoresis/Laser-Induced Fluorescence (CE-LIF)

Several CE-LIF systems with different characteristics were used to quantify the glutamate and 5-HT samples [52]. For most measurements, the glutamate was analyzed by derivatizing 5 μl of microdialysis sample with 1 μl of 3×10^−6^ fluorescein, 2 μl of 10 mM naphthalene-2,3-dicarboxaldehyde (NDA), 1 μl KCN, and 1 μl of 50 mM borate buffer (pH 9.4). NDA forms a stable fluorescent product with glutamate that can be measured by capillary electrophoresis/laser-induced fluorescence (CE-LIF) [53], except that the laser, focusing lens, and optics have been changed to a different excitation wavelength (Ex = 457.9 nm). Briefly, an Argon ion laser (Melles Griot; Carlsbad, CA) was employed to provide the 457.9 nm wavelength laser line and a microscope objective was used to focus the beam to a post-column sheath flow assembly. The fluorescence was collected and focused by an all-reflective microscope objective, and a focusing lens (f = 8.0 cm at 257 nm). A 488 nm notch filter, a 475 nm long-pass filter (03FCG465/GG475; Melles Griot; Carlsbad, CA), and a 550 nm short-pass filter (03SWP408; Melles Griot; Carlsbad, CA) were used to select the appropriate fluorescence signal. The micro-injector, high voltage power supply, sheath flow assembly, and PMT were the same as previously described [53]. The 50-μm I.D./360-μm O.D. uncoated fused-silica capillary (Polymicro Technologies; Phoenix, AZ) was 65–75 cm long. Relative concentrations for all samples were determined by analyzing the ratio of glutamate to fluorescein.

A distinct automated CE-LIF system was used for the glutamate measurements from the SCN after stimulation to the median raphe (as shown in Figure 3). Here the glutamate was analyzed by derivatizing 1 μl of microdialysis sample with 0.5 μl of 16 μM fluorescein (as an internal standard), 0.5 μl of 4 μM L-cysteic acid, 0.5 μl of 10 mM naphthalene-2,3-dicarboxaldehyde (NDA) in methanol, and 0.5 μl of 20 mM KCN. Fluorescein, L-cysteic acid, and KCN were dissolved in 100 mM borate buffer (pH 9.5). The mixture of the sample and the derivatization reagents was allowed for 45 min to complete the reaction in the dark at room temperature. After the derivatization, the mixture was diluted by adding 17 μl of 100 mM borate buffer (pH 8.7). An automated CE system, P/ACE MDQ with LIF detector (Beckman Coulter, Fullerton, CA), and 75-μm I.D./360-μm O.D. fused-silica capillary (Polymicro Technologies, Phoenix, AZ) was used. A diode laser emitting 442±7 nm (CVI Melles Griot, Carlsbad, CA) was used as the excitation source for LIF. Fluorescence signal was selected by a band-pass filter of 490±15 nm (Omega Optical, Brattleboro, VT) and was set before a photomultiplier tube of the LIF detector. The separation buffer consisted of 100 mM borate, 30 mM sodium deoxycholate, and 20 mM β-cyclodextrin at pH 8.7. The sample was injected by pressure of 0.5 psi for 5.0 sec and was separated by 27 kV normal polarity. Relative concentrations of glutamate for all samples were determined by analyzing the ratio of glutamate to fluorescein peak areas.

5-HT and its metabolites were identified and quantified using native fluorescence detection [54]. After thawing the samples, 1 µL of the microdialysate sample was diluted with 2.5 µL of 7.55 µM sulforhodamine-101, used as an internal standard, and 6.5 µL of ultrapure water (PURELAB Ultra Analytical; Siemens Water Technology; Warrendale, PA) for a total volume of 10 µL. Samples were analyzed using a custom-built capillary electrophoresis/laser-induced native fluorescence (CE-LINF) instrument [55]. Briefly, a 224 nm HeAg hollow cathode ion laser (HeAg70; Photon Systems; Covina, CA) is used to excite 5-HT and its metabolites, which are natively fluorescent. The incident beam is nominally focused to a 50 μm spot immediately below the outlet of the capillary, which is housed in a sheath flow cuvette. The inlet of the flow cell is connected to a sheath flow reservoir and the outlet is connected to a waste reservoir. A height difference is maintained between the liquid levels in these two reservoirs such that a linear sheath fluid velocity of ∼0.2 mm/s is generated.

Fluorescence is collected orthogonal to the excitation by a 15x all-reflective microscope objective (13596; Newport; Irvine, CA). The collimated beam then impinges upon two custom coated dichroic beamsplitters (Model and lot numbers 310dcxxr-haf #110258 and 400dcxru #111563; Chroma Technology; Rockingham, VT), with transitions at 310 nm and 400 nm, respectively. The signal is directed, based on wavelength, into one of three photomultiplier tubes (H6780-06; Hamamatsu; Middlesex, NJ), referred to as “blue” (250–310 nm), “green” (310–400 nm), and “red” (400^+^ nm). The 50-μm I.D./360-μm O.D. uncoated fused silica capillary (Polymicro Technologies; Phoenix, AZ) was 70 cm long. The inlet of the capillary was grounded and -30 kV (PS/MJ30N0400-11; Glassman High Voltage; High Bridge, NJ) was applied to the waste reservoir. The background electrolyte for all analyses was 40 mM borate buffer, pH 8.8, and the sheath flow solution was 25 mM citric acid, pH 2.4.

This instrument allows identification of analytes based on their spectral characteristics in addition to their migration times. Data were analyzed using in-house programming in Igor Pro 5.03 (WaveMetrics; Lake Oswego, OR). Electropherograms were normalized to laser intensity and smoothed using 6-point boxcar averaging. Quantitation was performed using equations derived from calibration curves in the appropriate concentration ranges for each analyte of interest.

### Circadian Wheel-running Behavior

For analyzing circadian state in mice, animals were individually housed in cages equipped with a running wheel. A magnet (neodinium grade-27; Magnetic Energies, Inc.; San Antonio, TX) was attached to each wheel, and each revolution of the wheel moved it past a hermetically sealed reed switch (HSR-067RT; Hermetic Switch, Inc.; Chickasha, OK), closing a circuit [48]. This circuit closure was transmitted to a computer running Clocklab Acquisition software (Actimetrics, Inc.; Evanston, IL). Data were stored in 6-min bins, and the resulting rhythm was plotted in an actogram and analyzed with Clocklab Analysis software (Actimetrics, Inc.; Evanston, IL).

Because of the need to include neostigmine in the perfusion fluid for microdialysis experiments, which could interfere with the circadian behavior of the animal, microdialysis experiments were performed separately from behavioral experiments. Animals were entrained to a 12-h L:12-h D cycle for at least 10 days following surgery, and then were released to constant darkness (DD). Following at least 10 days in DD, treatment times were calculated as described previously [48]. For stimulation experiments, 5 min before the predicted treatment time, the subject was removed under dim (<1 lux) red light, gently restrained, and a connecting cable (305-305 TT2(C); Plastics One; Roanoke, VA) was attached to the previously implanted electrode. The subject was returned to its home cage, and placed in one of two CAMS designated for stimulations. The connecting cable was attached to an electronic swivel (SL2C; Plastics One; Roanoke, VA) and the animal was stimulated (Grass SD9 Stimulator; Grass Product Group; West Warwick, RI) at the calculated CT of treatment. Sham animals were attached to the connecting cable/swivel for 20 min, but no current was passed through the stimulator. Immediately following stimulation, the sham subject was unhooked from the connecting cable and returned to home housing. For behavioral experiments, an individual mouse underwent up to 4 separate treatments, separated by at least 10 days, with the order of treatment times randomized between mice.

Activity data were analyzed using Clocklab Analysis software (Actimetrics, Inc.; Evanston, IL). Onset of activity was determined using Clocklab's template matching algorithm, and was confirmed visually. The magnitude of phase resetting was measured as the difference between regression lines drawn through the 5 days immediately before treatment and 5 days after reestablishment of a stable circadian rhythm after treatment. *Tau* (*τ*) was calculated before and after each treatment, with no significant difference seen following any of the treatment conditions (Table S1).

### Histology

Following experiments, animals were sacrificed and brains were frozen on dry ice. Brains then were sliced into 40 μm sections using a cryostat, and stained with cresyl violet to determine the placement of the microdialysis probe within the SCN, or with cholinesterase staining to verify the placement of the stimulating electrodes within the LDTg, PPTg, and MRN using standard procedures [56]. Representative brain sections demonstrating cannula and electrode placement are shown in Figure S2.

### Statistics

Data were analyzed with either two-way analysis of variance (ANOVA) with Holm-Sidak *post hoc* comparison, or for the PPTg injection experiments, where all animals received all treatment, by paired Student's *t*-test, using SigmaPlot analysis software (Systat Software, Inc.; San Jose, CA). The level of significance was p<0.05.

## Supporting Information

Figure S1 **Stimulating the MRN increases 5-HT release at the SCN, but not ACh or glutamate.** Stimulation of the MRN at ZT 15 with Condition 1 (**A**, 150 µA, 10 Hz, 2-msec pulse duration) or Condition 2 (**B**, 40 µA, 0.2 Hz, 0.2-msec pulse duration), does not significantly increase ACh, glutamate (Glu), or 5-HT levels at the SCN. However, stimulating the MRN with Condition 3 (**C**, 400 µA, 60 Hz, 0.2-msec pulse duration, applied as a 1-sec train every min/20 min) significantly increases 5-HT release at the SCN, without affecting ACh or Glu levels. MRN, median raphe nucleus; 5-HT, serotonin. * indicates p<0.05 by Two-Way ANOVA with Holm-Sidak *post-hoc* test.
(TIF)

Figure S2 **Coordinates and anatomy of electrode and cannula placements in mouse brains.** Pictured on the left of each panel are the relevant coronal planes of sterotaxic coordinates [47]. A box surrounds the approximate area of the histological image on the right. Arrows mark the path of probe/electrode. **A** is stained with cresyl violet, while **B–E** are stained for cholinesterase. All coordinates are based on distance from *bregma*. (**A**) SCN (AP -0.1, ML -0.1, DV -4.0). (**B**) Left: MRN electrode (AP -3.8, ML 0.0, DV -4.8), Right: PPTg electrode (AP -4.3, ML -1.8, DV -3.7). (**C**) LDTg (AP -5.2, ML -0.5, DV -3.3). (**D**) PPTg injection probe (AP -4.3, ML -1.8, DV -2.7).
(TIF)

Table S1 **Average** ***τ*** **values for mice before (pre-) and after (post-) treatment.** Analysis of the data with paired t-test revealed no significant difference in *τ* before *vs.* after treatment for any of the groups.
(TIF)
