# Supplementary figures and images for: Signals from the Brainstem Sleep/Wake Centers Regulate Behavioral Timing via the Circadian Clock
Source: PLoS One. 2013 Aug 12;8(8):e70481. doi: 10.1371/journal.pone.0070481 (PMC3741311; doi:10.1371/journal.pone.0070481)

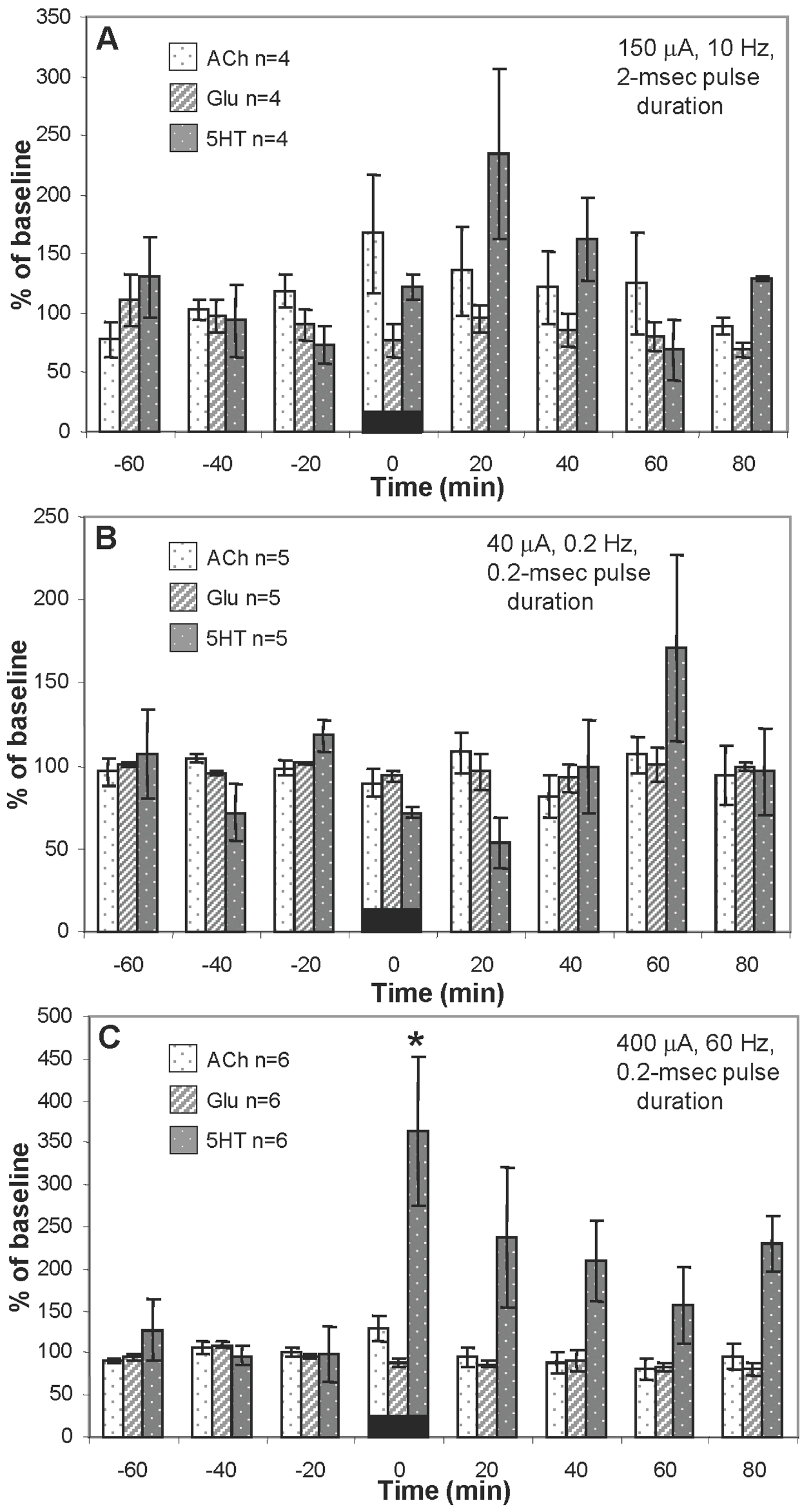

Supplement: Figure S1 — Stimulating the MRN increases 5-HT release at the SCN, but not ACh or glutamate. Stimulation of the MRN at ZT 15 with Condition 1 (A, 150 µA, 10 Hz, 2-msec pulse duration) or Condition 2 (B, 40 µA, 0.2 Hz, 0.2-msec pulse duration), does not significantly increase ACh, glutamate (Glu), or 5-HT levels at the SCN. However, stimulating the MRN with Condition 3 (C, 400 µA, 60 Hz, 0.2-msec pulse duration, applied as a 1-sec train every min/20 min) significantly increases 5-HT release at the SCN, without affecting ACh or Glu levels. MRN, median raphe nucleus; 5-HT, serotonin. * indicates p<0.05 by Two-Way ANOVA with Holm-Sidak post-hoc test. (TIF) [file pone.0070481.s001.tif]

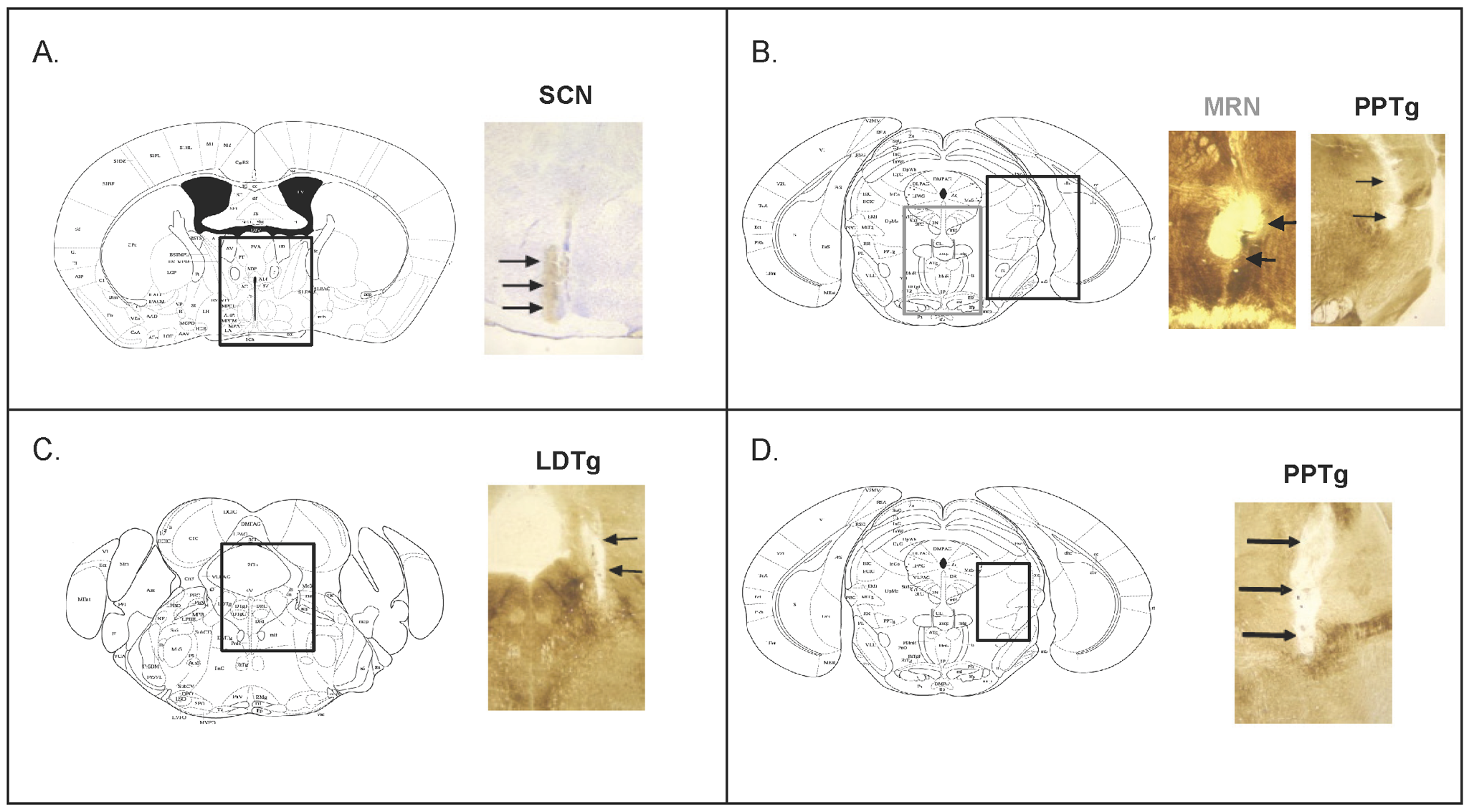

Supplement: Figure S2 — Coordinates and anatomy of electrode and cannula placements in mouse brains. Pictured on the left of each panel are the relevant coronal planes of sterotaxic coordinates [47]. A box surrounds the approximate area of the histological image on the right. Arrows mark the path of probe/electrode. A is stained with cresyl violet, while B–E are stained for cholinesterase. All coordinates are based on distance from bregma. (A) SCN (AP -0.1, ML -0.1, DV -4.0). (B) Left: MRN electrode (AP -3.8, ML 0.0, DV -4.8), Right: PPTg electrode (AP -4.3, ML -1.8, DV -3.7). (C) LDTg (AP -5.2, ML -0.5, DV -3.3). (D) PPTg injection probe (AP -4.3, ML -1.8, DV -2.7). (TIF) [file pone.0070481.s002.tif]

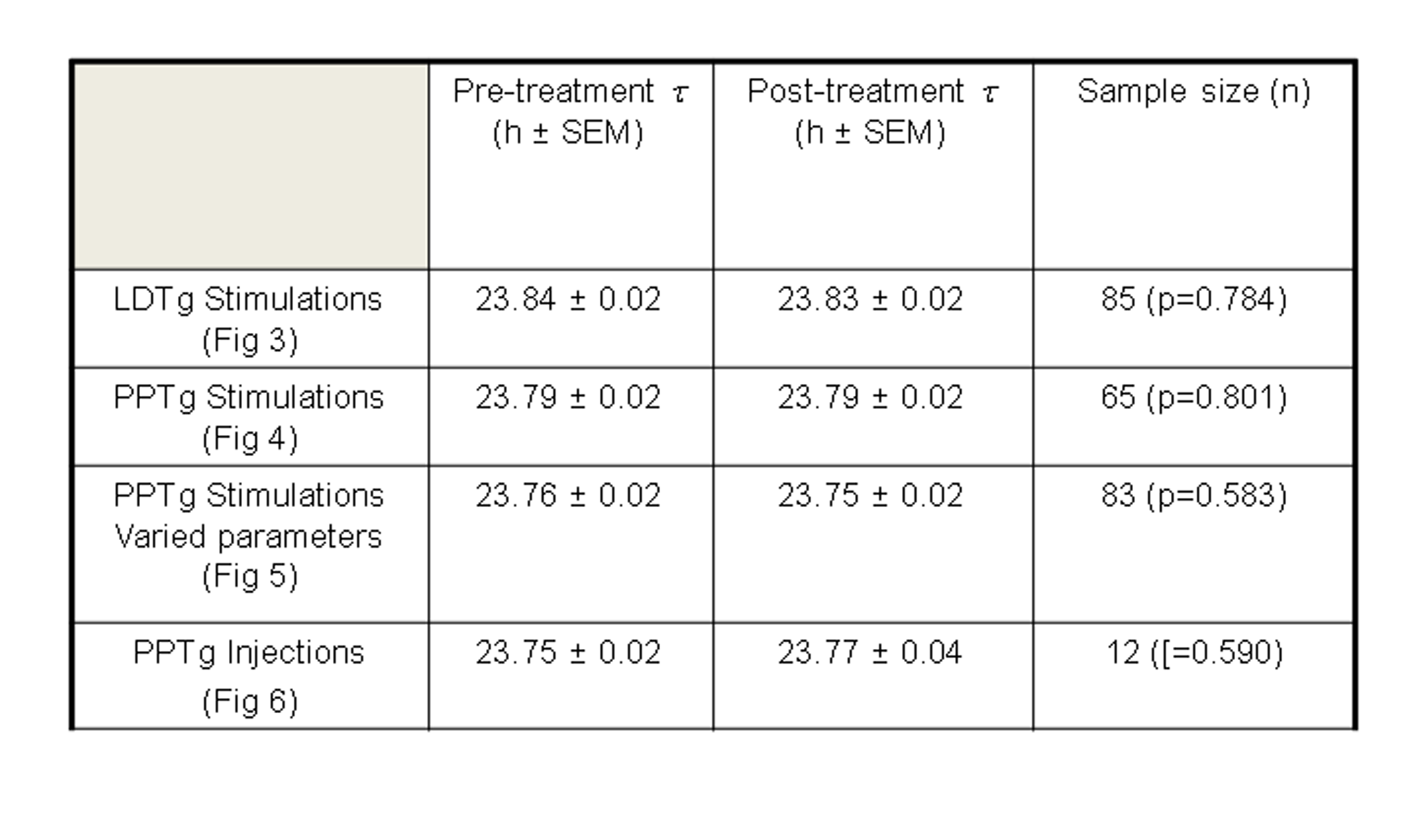

Supplement: Table S1 — Average τ values for mice before (pre-) and after (post-) treatment. Analysis of the data with paired t-test revealed no significant difference in τ before vs. after treatment for any of the groups. (TIF) [file pone.0070481.s003.tif]
